# The Relationship between Advanced Oxidation Protein Products, Vascular Calcifications and Arterial Stiffness in Predialysis Chronic Kidney Disease Patients

**DOI:** 10.3390/medicina57050452

**Published:** 2021-05-06

**Authors:** Ion-Vlad Vinereanu, Ileana Peride, Andrei Niculae, Andreea Taisia Tiron, Andra Caragheorgheopol, Dana Manda, Ionel Alexandru Checherita

**Affiliations:** 1 Clinical Department No. 3, “Carol Davila” University of Medicine and Pharmacy, 050474 Bucharest, Romania; vlad.vinereanu@gmail.com (I.-V.V.); al.checherita@gmail.com (I.A.C.); 2 Department of Cardiology, “Carol Davila” University of Medicine and Pharmacy, 050474 Bucharest, Romania; taisia_andreea@yahoo.com; 3 Department of Endocrinology, “C.I. Parhon” National Institute of Endocrinology, 011863 Bucharest, Romania; andracaragheor@yahoo.com (A.C.); danaa.manda@yahoo.com (D.M.)

**Keywords:** CKD, arterial stiffness, oxidative stress, AOPP, vascular calcification

## Abstract

*Background and Objectives*: Cardiovascular morbidity and mortality are increased in patients with chronic kidney disease (CKD). It is likely that the accumulation of uremic toxins resulting in increased oxidative stress (OS) is a major contributing factor, but no clear link has been identified. The purpose of this research is to establish if advanced oxidation protein product (AOPP) levels in the serum of predialysis patients are a contributing factor to vascular calcification and increased arterial stiffness. *Materials and Methods*: After obtaining the informed consent, 46 predialysis patients (CKD stages G3–G5) were included in the study. In order to identify vascular calcifications, hand and pelvic radiographs were performed. Valvular calcifications were identified using cardiac ultrasound. AOPP were measured using a commercially available ELISA kit. The relationships between serum AOPP values and biochemical parameters relevant in the evaluation of CKD patients were analyzed. In addition to identifying the differences in AOPP levels between patients with/without vascular or valvular calcifications, the research focused on describing the relationship between OS and arterial stiffness assessed by oscillometric pulse-wave velocity (PWV) measurement. *Results*: No significant relationship between serum AOPP and vascular or valvular calcifications was highlighted, but significant correlations of AOPP with C-reactive protein (*p* = 0.025), HDL-cholesterol levels (*p* = 0.04), HbA1c (*p* = 0.05) and PWV values (*p* = 0.02) were identified. *Conclusions*: The usefulness of (OS) measurement in clinical practice remains debatable; however, the relationship between AOPP and arterial stiffness could be valuable in improving cardiovascular risk assessment of patients with CKD.

## 1. Introduction

Oxidative stress (OS) is a key pathological feature of chronic kidney disease (CKD) that results, partly, from prolonged exposure to uremic toxins and contributes to patient morbidity and mortality. Until recently, the quantification of reactive oxygen species (ROS) could only be performed in vitro studies, because ROS have very short half-lives and usually react with other molecules near the site where they are produced, making it difficult to isolate and adequately analyze them [1]. Free radicals oxidize lipids, proteins and nucleic acids, producing more stable compounds that can be assessed as biological markers of OS [1].

Currently, the available markers indirectly evaluate the levels of ROS and are not able to identify the source of free radicals [2]. The pathologies most frequently associated with CKD (i.e., diabetes mellitus, hypertension, atherosclerosis) are themselves major disruptors of redox homeostasis and prevent researchers from adequately evaluating the role of ROS in CKD pathogenesis [3]. These assessments could provide valuable insight regarding the severity and progression of CKD and its complications.

Among the markers relevant for CKD are: malondialdehyde (MDA), thiobarbituric acid reactive substances (TBARS), F2-isoprostanes, lipid hydroperoxides (LOOH), asymmetric dimethylarginine (ADMA), homocysteine, protein carbonyls and advanced oxidation protein products (AOPP) [2].

AOPP result from albumin exposure to chlorinated oxidants that result during macrophage or neutrophil activation, and can be found in the serum of CKD patients as dityrosine or disulfide protein aggregates [4,5].

AOPP levels correlate with CKD severity and progression and vary according to dialysis modality, with hemodialysis patients having the highest concentrations [4]. As Cao et al. have shown, AOPP are biologically active molecules that can contribute to kidney disease progression by local activation of the renin–angiotensin–aldosterone axis, and exhibit indirect oxidant activity by the stimulation of NADPH-oxidase in proximal tubular cells [6].

Zhou et al. concluded that AOPP promote podocyte apoptosis by interacting with the receptor for advanced glycation-end products (RAGE) with subsequent NADPH-oxidase activation. Podocytes exposed to oxidized albumin express molecules correlated with the p53/Bax/caspase-3 signaling pathway (associated with apoptosis) [7].

Descamp-Latscha et al. have demonstrated that AOPP and acute phase reactants (i.e., C-reactive protein and fibrinogen) have a significant predictive capacity for adverse cardiovascular events in predialytic CKD patients [8]. Among type 2 diabetic patients, elevated levels of AOPP were associated with endothelial dysfunction caused by depletion of vasodilator substances (i.e., nitrous oxide) and impaired adaptation of small vessel caliber to tissue perfusion requirements [9], with possible negative effects on the process of arteriovenous fistula maturation [10].

Vascular calcification can be defined as the pathological process through which minerals (i.e., calcium phosphate salts) actively deposit within the layers of the vascular walls, frequently associated with aging, diabetes, cardiovascular disease and CKD [11]. Generally, two types of vascular calcifications have been recognized: intimal and medial. Intimal vascular calcifications are encountered both in CKD patients and in the general population, and usually manifest in the form of atherosclerotic plaques that can be entirely calcified and less prone to rupture, or plaques with microcalcifications that are more likely to rupture and cause thromboembolic events [12]. Irrespective of the level of calcification, atherosclerotic plaques are usually associated with luminal obstruction and impaired organ perfusion [13]. In CKD, atherosclerotic plaques evolve at an accelerated rate, owing to the various metabolic disturbances and associated comorbidities [14].

Medial calcifications occur by abnormal calcium phosphate deposition in the arterial media and through subsequent changes in vascular smooth muscle cells (VSMCs) phenotypes under the influence of inflammatory cells and mediators [15,16,17,18]. This type of pathological mineralization is frequently encountered in patients with CKD and is more pronounced in dialysis-dependent end-stage renal disease, in part as a consequence of dysregulated calcium phosphate metabolism [15,19]. Unlike intimal calcifications, medial calcification does not obstruct the vascular lumen, but it promotes arterial stiffening, increased pulse pressure and increased shear-stress [15].

AOPP seem to favor vascular mineralization by acting upon VSMCs, inducing the transition to an osteoblastic phenotype. VSMCs have increased cytosolic calcium concentrations and express elevated levels of CBF-α1 (core binding factor) mRNA involved in the synthesis of osteocalcin and alkaline phosphatase [20]. There are reports that confirmed that AOPP promote cardiomyocyte apoptosis and defective cardiac remodeling in CKD by activating the JNK pathway (c-Jun N-terminal kinase pathway) [21]. Lin et al. have identified increased levels of AOPP in patients with coronary calcifications [22].

Many of the studies involving the effects of AOPP in CKD have been performed in vitro; these studies did not focus on analyzing AOPP as a useful clinical marker. Therefore, the aim of the present study was to investigate the relationship between AOPP, vascular calcifications and other cardiovascular assessment tools, routinely used for the evaluation of CKD patients.

## 2. Materials and Methods

This research represents a cross-sectional study performed on a prevalent cohort of Caucasian inpatients with CKD stages G3–G5 admitted between July 2018 and January 2019 in the Department of Nephrology of an emergency hospital for routine evaluation and not undergoing hemodialysis at the time of inclusion. Most of the patients included were diagnosed with hypertension, diabetes or both, were undergoing chronic treatment for these pathologies and were hemodynamically stable and had relatively adequate glycemic control at the time of inclusion. Exclusion criteria were: cerebrovascular events during the 6 months prior to the inclusion in the study (i.e., ischemic or hemorrhagic stroke, acute myocardial infarction), severe heart failure, acute hepatitis or liver failure, cirrhosis, active neoplasia and patients on chronic hemodialysis. The study was approved by the Hospital Local Ethics Committee and was conducted in accordance with the Declaration of Helsinki.

### 2.1. Demographic and Clinical Parameters

After the informed consent was obtained, information regarding age, gender, history of CKD disease, the presence of diabetes mellitus, arterial hypertension, coronary and peripheral artery disease, as well as other comorbidities were retrieved from patients’ medical records. At the moment of inclusion, the patients were weighed and measured. Systolic and diastolic blood pressures were measured at the upper arm after 10 min of rest, and pulse-wave analysis was performed using Mobil-O-Graph^®^ NG, offering information regarding pulse-wave velocity (PWV), augmentation index (AIX) and augmentation pressure (AugP).

### 2.2. Laboratory Parameters

Blood samples were taken, assessing: complete blood count, glycemia, glycated hemoglobin for diabetic patients (HbA1c), serum urea, creatinine and uric acid, full lipid profile (total cholesterol, LDL-cholesterol (low-density lipoprotein cholesterol), HDL-cholesterol (high-density lipoprotein), triglyceride), inflammatory markers (erythrocyte sedimentation rate (ESR), fibrinogen, C-reactive protein (CRP)), phosphocalcic metabolism parameters (total serum calcium, serum phosphate, intact parathormone (iPTH)), total serum proteins, and albumin, proteinuria and albuminuria. Estimated glomerular filtration rate (eGFR) was calculated using the CKD-EPI formula [23]. Analyses were performed by the accredited hospital laboratory. For AOPP evaluation: blood was drawn in 2 mL EDTA tubes and centrifuged for 15 min at 7000 RPM, then the plasma was transferred into plain test tubes and stored at −24 °C. The samples were analyzed by a spectrophotometric method using a commercially available AOPP kit (Immundiagnostik, Bensheim, Germany). Samples were prepared according to manufacturer’s instructions: the samples and reagents were brought to room temperature (20–30 °C), and the EDTA-plasma samples were centrifuged in 1.5 mL test tubes at 3000× *g* for 30 s. Then, 125 μL of centrifuged EDTA-plasma was treated with 25 μL of delipidation reagent and left to incubate for 10 min at room temperature. After centrifuging again for 5 min at 3000× *g*, 100 μL delipidated EDTA-plasma was mixed with 400 μL assay buffer, resulting in a final dilution of 1:6. The absorbance of the standards, controls and patient samples was read at 340 nm, expressing AOPP concentrations as chloramine T(CT)-equivalents. The resulting AOPP concentration was multiplied by a dilution factor of 6.

### 2.3. Imaging Studies

For valvular calcification assessment: a Doppler echocardiogram was performed (by the same operator) using a Samsung HS40 system with a 4 GHz Samsung Medison PN2-4 probe (Samsung Medison, Seoul, Korea) for cardiac ultrasound (a standardized protocol was followed to minimize result variability). The focus of the examination was on the mitral and aortic valves.

To detect vascular calcifications, hand and pelvic radiographs were performed, and the films were analyzed by the same operator using the protocol described by Adragao et al.: the pelvic radiograph was divided into 4 quadrants by two imaginary lines, a horizontal line just above the femoral heads and a vertical line over the middle of the vertebral column; the hand radiographs were divided similarly by a horizontal line over the upper limit of the metacarpal bones and one vertical line between the two hands [24]. If linear calcifications corresponding to medial calcification of the arteries were noted in any of the 4 quadrants, the patient received 1 point for every quadrant with visible vascular calcifications; the score was the sum of positive quadrants, with a minimum of 1 and a maximum of 8.

### 2.4. Statistical Analysis

Database management and statistical analysis were performed using IBM SPSS 20. Continuous variables were tested for normality by visually inspecting the frequency distribution plots and by performing the Shapiro–Wilk test. Non-normally distributed data were log-transformed in order to meet the assumptions of normality for parametric testing. Dispersion of continuous variables were characterized using means and standard deviations (SDs), while those that were not normally distributed were described by their medians and interquartile ranges (IQR). To assess AOPP, Pearson’s chi-square test was used to compare dichotomous variables for significant differences. To evaluate the differences between patients with and without vascular/valvular calcifications, an independent samples t-test was used (boxplots). A hierarchical multiple linear regression analysis was performed to examine the relationship between AOPP and vascular stiffness.

## 3. Results

### 3.1. Patient Characteristics

**Figure 1:**
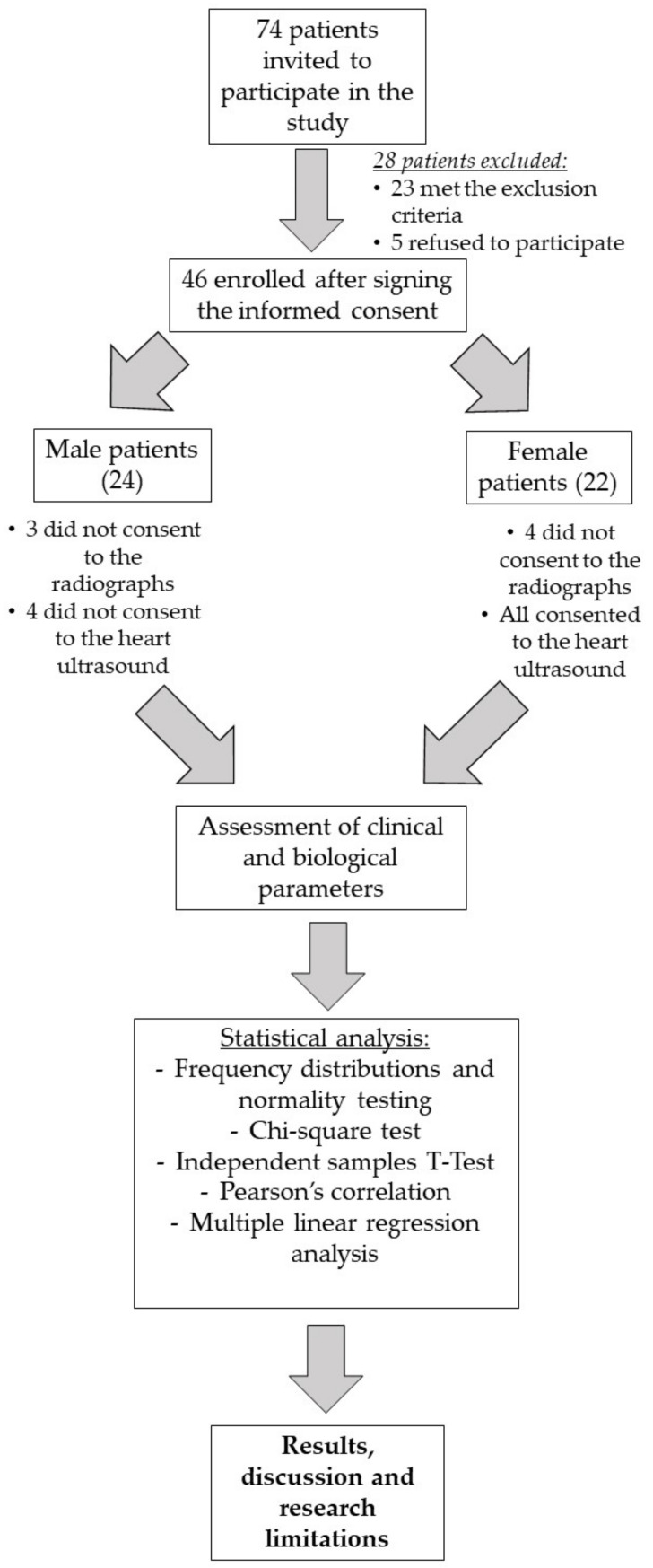
Design of the study.

74 patients diagnosed with chronic kidney disease (CKD) stages G3–G5 were invited to participate in this study, but after applying the inclusion and exclusion criteria, a total of 46 patients were enrolled: 22 females and 24 males, with a mean age of 65.07 ± 13.89 years and a median glomerular filtration rate of 10 mL/min/1.73 m^2^ (IQR = 9.86, IQR = interquartile range). Of these, 78.3% were diagnosed with arterial hypertension and 43.5% with diabetes mellitus. Seven patients did not consent to the radiographs (84.7% consent rate), and 4 did not consent to the Doppler echocardiogram (91.3% consent rate). AOPP values ranged from 9.9 to 45.78 µmol/L, with a slightly higher level in females than in males (Table 1 and Figure 1).

### 3.2. Vascular and Valvular Calcifications

Of the 46 patients included in the study, 29 (63%) presented vascular calcifications visible on the performed radiographs, 24 (52.1%) had valvular calcifications seen on cardiac ultrasound, 16 had both vascular and valvular calcifications and 2 had none. There were no statistically significant differences between genders regarding vascular (*n* = 39, χ^2^ = 0.07, *p* = 0.9) and valvular calcifications (*n* = 42, χ^2^ = 0.07, *p* = 0.791). There were no differences in mean age between the patients that had (*M1*) or did not have (*M2*) vascular calcifications (*M1* = 67.7 ± 13.9 yrs. vs. *M2* = 62.2 ± 16.15 yrs., *t(37)* = 1.051, *p* = 0.3) or valvular calcifications (*M1* = 69.3 ± 14.39 yrs. vs. *M2* = 61.44 ± 12.64 yrs., *t(40)* = 1.850, *p* = 0.07) (Table 2).

**Figure 2:**
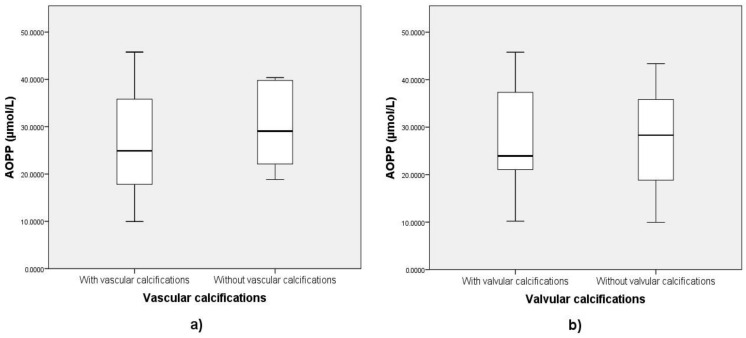
Differences in AOPP values between patients with and without vascular calcifications visible on (**a**) pelvic and hand radiographs and (**b**) cardiac ultrasound. Legend: AOPP = advanced oxidation protein products.

Mean AOPP values were not significantly different between groups with or without vascular calcifications (*M1* = 25.96 ± 10.9 µmol/L vs. *M2* = 29.8 ± 8.58 µmol/L, *t(37)* = −1.013, *p* = 0.31) or valvular calcifications (*M1* = 27.3 ± 10.3 µmol/L vs. *M2* = 26.9 ± 10.07 µmol/L, *t(40)* = 0.136, *p* = 0.89) (Figure 2a,b, Table 2).

### 3.3. Pulse-Wave Analysis and Arterial Stiffness

**Figure 3:**
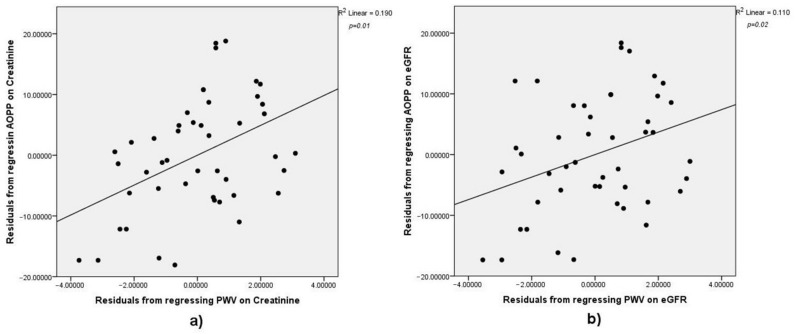
Scatterplots illustrating the positive correlation between PWV (m/s) and AOPP (µmol/L) after adjusting for the effect of serum creatinine (**a**) and eGFR (**b**). As shown by the differences in the r-value, serum creatinine improved the relationship between AOPP and PWV more than eGFR. Legend: PWV = pulse wave velocity; AOPP = advanced oxidation protein products; eGFR = estimated glomerular filtration rate.

Mean systolic blood pressure was significantly higher in men (*MsysM*) than in women (*MsysF*): *MsysM* = 144 ± 15.71 mmHg, *MsysF* = 129 ± 16.56 mmHg, *t(44)* = 3.075, *p* = 0.004. Mean arterial pressure was significantly higher in men (*MmapM*) than in women (*MmapF*): *MmapM* = 109.29 ± 11.74 mmHg, *MmapF* = 98.5 ± 12.56 mmHg, *t(44)* = 3.012, *p* = 0.004; however, it had no relationship with serum AOPP (*r* = −0.16, *p* = 0.13) or PWV (*r* = −0.31, *p* = 0.42). There were no significant differences regarding PWV values between male (*MpwvM*) and female (*MpwvF*) gender (*MpwvM* = 9.81 ± 1.86 m/s, *MpwvF* = 9.24 ± 1.85 m/s, *t(44)* = 1.042, *p* = 0.303). Zero-order correlation between AOPP and PWV was statistically significant but rather weak (*n* = 46, *r* = 0.29, *p* = 0.02). Performing the analysis while controlling for the effects of eGFR (*n* = 46, *r* = 0.332, *p* = 0.02) and creatinine (*n* = 46, *r* = 0.43, *p* = 0.01) revealed that serum creatinine levels had a greater influence on the relationship between PWV and AOPP than eGFR (Figure 3a,b). Unlike PWV, pulse pressure did not correlate with AOPP (*r* = 0.13, *p* = 0.19).

To identify the strongest PWV predictors, 3 linear regression models were tested using eGFR and AOPP as common independent variables and adding one of the three additional variables (systolic blood pressure (SysBP), pulse pressure (PP), augmentation pressure (AugP)) to avoid multiple collinearity (Table 3). As shown, the model including the pulse pressure explained 34% of PWV variability in the studied population, with PP (*β* = 0.46) and AOPP (*β* = 0.24) being the largest contributors to the model.

Fourteen patients exhibited isolated systolic hypertension, defined as systolic blood pressure > 140 mmHg and diastolic pressure below 90 mmHg, without a notable difference in AOPP levels (*t(44)* = −0.445, *p* = 0.658). The statistical analysis revealed significantly higher proteinuria (*t(37)* = 2.67, *p* = 0.01) and PWV values (*t(44)* = 2.16, *p* = 0.036).

### 3.4. Metabolic Parameters and their Relationship with AOPP

Statistical analysis of the relationship between AOPP levels and various metabolic parameters used in the routine evaluation of patients with CKD is outlined in Table 4. Apparently, AOPP levels do not correlate with serum creatinine, serum urea, uric acid or eGFR (Table 4).

**Figure 4:**
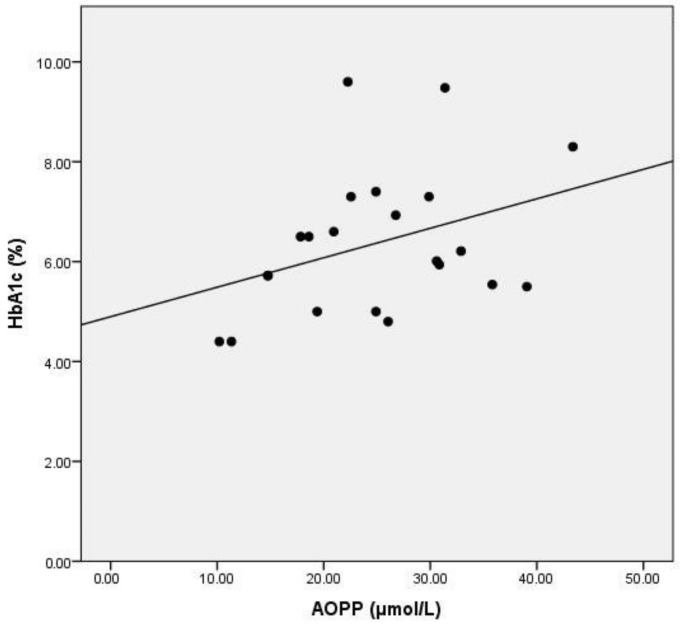
Correlation between AOPP and glycated hemoglobin values. Legend: AOPP = advanced oxidation protein products.

**Figure 5:**
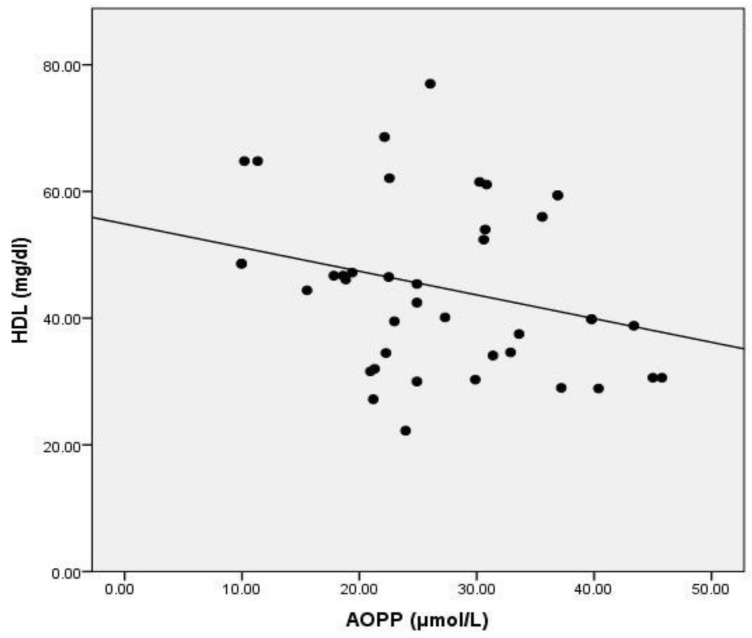
Correlation between AOPP and HDL-cholesterol. Legend: AOPP = advanced oxidation protein products; HDL = high-density lipoprotein.

**Figure 6:**
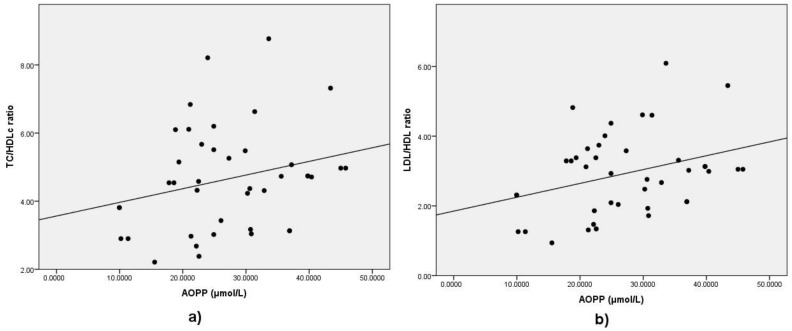
Correlation between AOPP and (**a**) TC/HDL-cholesterol ratio, and (**b**) LDL/HDL-cholesterol ratio. Legend: AOPP = advanced oxidation protein products; TC = total cholesterol; HDL = high-density lipoprotein; LDL = low-density lipoprotein.

Serum AOPP did not correlate with glycemia levels, but exhibited a positive correlation with the value of glycated hemoglobin (Table 4, Figure 4). Total cholesterol (TC) and LDL-cholesterol levels did not exhibit any significant relationship to AOPP. HDL cholesterol inversely correlated with serum AOPP levels (Table 4, Figure 5). The total cholesterol/HDLc and LDLc/HDLc (LDL-cholesterol/HDL-cholesterol) ratios positively correlated with serum AOPP (Table 4, Figure 6a,b). Neither serum calcium nor phosphate levels correlated with AOPP in the studied population sample.

### 3.5. Relationship between Serum AOPP and Inflammation Markers

**Figure 7:**
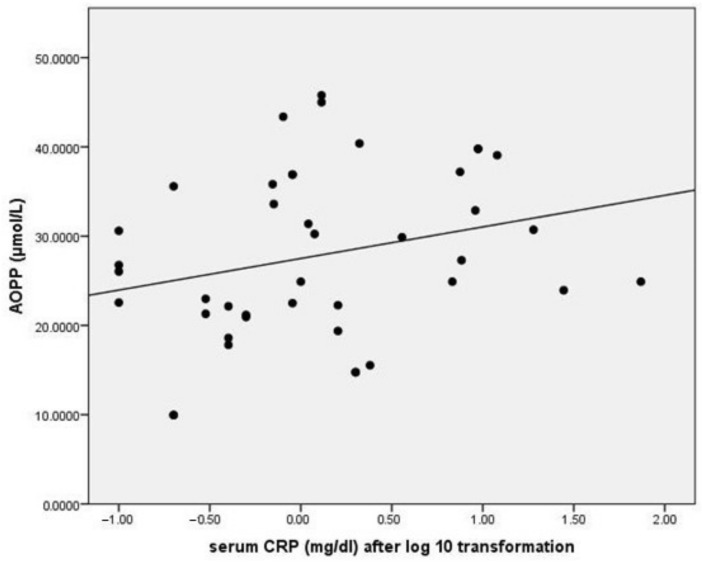
Correlation between AOPP and serum C-reactive protein (after log-10 transformation). Legend: AOPP = advanced oxidation protein products.

AOPP correlated with C-reactive protein values but not with the other markers (Table 4, Figure 7). Neutrophil-to-lymphocyte ratio (NLR) exhibited a positive correlation with age, PWV, PCR and platelet-to-lymphocyte ratio (PLR). PLR correlated with NLR and erythrocyte sedimentation rate. There were no significant differences between patients with or without vascular/valvular calcifications regarding the mentioned inflammation markers.

## 4. Discussion

In summary, our analysis of the CKD-population sample revealed the following: AOPP levels did not differ significantly among patients with/without vascular or valvular calcifications; AOPP and PWV were positively correlated, even more so when adjusting for serum creatinine levels; and AOPP levels and pulse pressure were significant predictors of PWV. Furthermore, we found no correlation between AOPP and serum creatinine or eGFR. HbA1c and HDL-cholesterol, but not LDLc and total cholesterol, along with the lipid ratios (TC/HDLc and LDLc/HDLc), positively correlated with serum AOPP levels.

Systemic vascular calcifications are an important determinant of poor cardiovascular outcomes in patients with CKD; however, the relationship with uremic toxins and OS has yet to be highlighted in clinical practice.

The influence of OS and AOPP on vascular/valvular calcification development has been underlined in several studies over time, but only in experimental ones [25,26,27]. There are still some questions regarding the correlation between AOPP and other clinical or biological parameters routinely associated with poor cardiovascular outcomes (arterial stiffness, lipid profile, markers of inflammation etc.); to our knowledge, no other investigators have studied these associations.

In the analyzed population sample, only 74.3% of patients had visible vascular calcifications on the performed radiographs, which was surprising, considering that most of our patients were CKD stages G4 and G5; medial calcifications probably were not as visible on plain radiographs as would be expected in predialysis patients. It is likely that the score proposed by Adragao et al. can be useful only in hemodialysis patients [24]. Of the included patients, 57% had visible valvular calcifications.

When assessing AOPP, no statistically significant differences were noted in patients with and without vascular calcifications. These results suggest that this OS marker is not related to the development of visible medial calcifications on radiographs or valvular calcifications on echocardiography. It is likely that the pathways linking OS and development of vascular medial calcifications do not depend nor relate to the generation of AOPP. Studies like the one performed by Gryszczyńska et al. demonstrated a significant increase of AOPP levels in patients with aneurysms of the abdominal aorta and aortoiliac occlusive disease [28]. Lin et al. have shown a marked increase of AOPP in patients with coronary artery disease and calcifications of the abdominal aorta [22]. Several differences exist between these studies and ours: in the study by Gryszczyńska et al., patients diagnosed with diabetes were excluded. Invasive computed tomography to identify the vascular calcifications was performed in that research, similar to the study by Lin et al. In order to reduce overall costs, we opted for plain radiographs and ultrasounds, as some authors have reported that ultrasound is as effective as computed tomography in identifying valvular calcifications, as long as the operator is experienced [29].

Aside from the accelerated development of atherosclerotic plaques in CKD, another feature is medial sclerosis of the arteries, which contributes to loss of wall elasticity and is associated with poor cardiovascular outcomes [30,31,32]. In our study, the relationship between AOPP and PWV was linear and statistically significant, unlike PP, which surprisingly did not correlate with AOPP. Performing the analysis while accounting for the effects of serum creatinine and eGFR showed that both variables improved the strength of the AOPP–PWV relationship, though it is likely that eGFR is a more relevant parameter in this context, since it better describes the renal function, unlike serum creatinine alone, which can be influenced by other factors (i.e., muscle mass) that have no known bearing on the amount of oxidative stress. Our linear regression model showed that pulse pressure, augmentation pressure, systolic blood pressure, AOPP and eGFR are significant predictors of PWV magnitude, with pulse pressure being the biggest contributor to PWV variability alongside eGFR and AOPP. The association between OS and arterial stiffness was expected, as in vitro studies have shown that reactive oxygen species induce phenotypical changes in vascular smooth muscle cells so that they lose their contractile properties and undergo a process similar to osteogenesis [25,33].

DeLeeuw et al. have shown that patients with isolated systolic hypertension, a feature of arterial stiffness, and altered renal function are at risk for significant cardiovascular morbidity and mortality [34]. In our study, we noticed that even if 24-hour proteinuria and PWV levels were significantly higher in patients with isolated systolic hypertension, there were no significant differences in AOPP levels between patients with and without isolated systolic hypertension.

We found no significant correlation of AOPP with eGFR or serum creatinine. There is a solid biological foundation for this relationship, since the accumulation of uremic toxins leads to an increase in the amount of generated reactive oxygen species [35,36,37]. Information regarding the relationship between AOPP specifically and renal function parameters is sparse and conflicting, with some authors reporting a positive relationship between AOPP and creatinine, while others found no such correlation. The discrepancies among studies result from a multitude of factors: relatively small sample sizes, differences in patient selection criteria, various metabolic disturbances inherent to additional comorbidities that are unaccounted for, differences in reagent characteristics etc. [38,39,40,41,42,43].

We analyzed the relationship between AOPP and total cholesterol, HDL-cholesterol and LDL-cholesterol in order to establish whether an atherogenic lipid profile was associated with increased OS in patients with CKD. We found a negative correlation between the levels of serum AOPP and HDL-cholesterol, suggesting that AOPP could be associated with inadequate reverse cholesterol transport and atheroma formation. Significant positive correlations with lipid ratios underline the relationship between OS and atherogenesis.

Regarding the relationship between AOPP and inflammatory markers, the Pearson correlation analysis revealed a linear relationship with C-reactive protein, but not with the other markers. Interestingly enough, our study identified a significant correlation between NLR and PWV, a finding that has been reported by other authors as well [44]. This suggests that a state of chronic inflammation can contribute to alterations in vascular wall elasticity, which can lead to poor cardiovascular outcomes.

### Limitations

While identifying several significant correlations between AOPP levels and routinely used biochemical parameters, the associations were relatively weak because of the small sample size. Another limitation was that our research was conducted in an emergency hospital, where most of the included subjects associated a complex pathology and a decreased eGFR (CKD stages G4 and/or G5).

## 5. Conclusions

Although the relationship between chronic inflammation and poor vascular health has long been established in CKD patients, the role of oxidative stress as a possible mediator of this relationship has not been sufficiently characterized. The specific OS markers in CKD have only been studied in vitro and seem to provide little useful information in clinical practice. While attempting to show the relevance of AOPP in clinical practice, this study identified a potentially valuable link between AOPP, PWV and several markers of inflammation, underlying the potential benefit of their use in cardiovascular health assessment, although further studies are required for adequate validation.

## Figures and Tables

**Table 1:** Patients’ demographic characteristics, and clinical and biochemical parameters.

Patients’ Characteristics		Value
Gender (%)	Male	52.2
	Female	47.8
Mean age (y)	65.07 (SD = 13.89)	
Smoking status (%)	Current smoker	23.9
	Nonsmoker	76.0
Arterial hypertension (%)	Hypertensive	78.3
	Non-hypertensive	21.7
Type 2 diabetes mellitus (%)	Diabetic	43.5
	Nondiabetic	56.5
Vascular calcifications (%)	With calcification	74.3
	Without calcification	25.6
Valvular calcifications (%)	With calcification	57.1
	Without calcification	42.8
**Pulse-Wave Analysis**		
PWV (m/s, M ± SD)		9.52 ± 1.83
Median augmentation pressure (mmHg)		9.5
IQR (mmHg)		16.25
Mean augmentation index (%, M ± SD)		25.3 ± 14.6
Mean pulse pressure (mmHg, M ± SD)		56.6 ± 18.6
**Advanced Oxidation Protein Products (µmol/L)**		
Male (M ± SD)		26.4 ± 8.4
Female (M ± SD)		27.6 ± 11.09
Total (M ± SD)		26.9 ± 9.7
**Renal Function Tests**		
Median serum creatinine (mg/dL)		5.0
IQR (mg/dL)		2.65
Serum urea (mg/dL, M ± SD)		142.0 ± 52.52
Median serum uric acid (mg/dL)		7.22
IQR (mg/dL)		3.40
Median eGFR (mL/min/1.73 m^2^)		10.0
IQR (mL/min/1.73 m^2^)		9.86
**Calcium Phosphate Metabolism**		
Serum calcium (mg/dL, M ± SD)		9.28 ± 0.77
Serum phosphate (mg/dL, M ± SD)		4.68 ± 1.07
iPTH (pg/mL, Median)		184.0
IQR (pg/mL)		230.2
Ca × PO_4_^−^ (mg^2^/dL^2^, M ± SD)		42.9 ± 9.8
**Lipid Profile**		
Cholesterol (mg/dL, M ± SD)		186.0 ± 53.1
LDL-cholesterol (mg/dL, M ± SD)		122.7 ± 42.85
HDL-cholesterol (mg/dL, M ± SD)		44.7 ± 13.2
Total cholesterol/HDLc ratio (M ± SD)		4.65 ± 1.5
LDLc/HDLc ratio (M ± SD)		2.92 ± 1.17
**Markers of Inflammation**		
Erythrocyte sedimentation rate (mm/h, M ± SD)		59.13 ± 37.2
Fibrinogen (mg/dL, M ± SD)		503.8 ± 152.6
Mean C-reactive protein (mg/L, M ± SD)		5.05 ± 12.22
**Other Tests**		
Serum albumin (mg/dL, M ± SD)		3.83 ± 0.78
Serum total proteins (mg/dL, Median)		7.11
IQR (mg/dL)		1.35
Albuminuria (mg/24 h, Median)		819.6
IQR (mg/24 h)		1881.94
Proteinuria (mg/24 h, Median)		776.0
IQR (mg/24 h)		1153.4

Legend: y = years; SD = standard deviation; PWV = pulse-wave velocity; M = mean value; IQR = interquartile range; eGFR = estimated glomerular filtration rate; iPTH = intact parathyroid hormone; Ca = serum calcium; PO_4_^−^ = serum phosphate; LDL = low-density lipoprotein; HDL = high-density lipoprotein; HDLc = HDL-cholesterol; LDLc = LDL-cholesterol.

**Table 2:** Patients’ characteristics outlined according to the presence/absence of vascular or valvular calcifications.

	Vascular Calcifications			Valvular Calcifications		
	Present	Absent	*p*-Value	Present	Absent	*p*-Value
Gender (Male/Female)	13/6	6/4	0.79	13/11	10/8	0.92
Age (M ± SD, y)	67.7 ± 13.9	62.2 ± 16.15	0.6	69.3 ± 14.3	61.4 ± 12.6	0.07
Diabetes mellitus (%)	14 (70%)	4 (25%)	0.65	10 (50%)	9 (45%)	0.59
Hypertension (%)	22 (61%)	8 (22%)	0.78	19 (52%)	14 (38%)	0.91
AOPP (M ± SD)	25.9 ± 10.9	29.8 ± 8.5	0.31	27.3 ± 10.3	26.9 ± 10.07	0.89
PWV (M ± SD)	9.8 ± 1.8	9.04 ± 1.9	0.24	10.04 ± 1.83	9.06 ± 1.7	0.08

Legend: M = mean value; SD = standard deviation; y = years; AOPP = advanced oxidation protein products; PWV = pulse wave velocity.

**Table 3:** Linear regression models for the prediction of PWV.

					Model Correlation Statistics		
		Standardized β	*T*-Value	*p*-Value	*r*-Value	*F*-Value	*p*-Value
MODEL 1	AOPP	0.316	2.306	0.02	0.446	4.858	0.03
	eGFR	0.336	2.457	0.018			
MODEL 2	AOPP	0.245	2.003	0.05	0.62	8.87	<0.01
	eGFR	0.20	1.579	NS			
	PP	0.466	3.605	0.01			
MODEL 3	AOPP	0.318	2.4	0.01	0.537	5.671	0.002
	eGFR	0.283	2.1	0.03			
	SysBP	0.304	2.3	0.02			
MODEL 4	AOPP	0.27	2.05	0.04	0.539	5.720	0.02
	eGFR	0.35	2.684	0.06			
	AugP	0.306	2.327	0.02			

Legend: AOPP = advanced oxidation protein products; eGFR = estimated glomerular filtration rate; NS = not statistically significant; PP = pulse pressure; SySBP = systolic blood pressure; AugP = augmentation pressure.

**Table 4:** Correlations between AOPP levels and various metabolic serum parameters or markers of acute inflammation.

	Pearson’s R/Spearman’s Rho *	*p*-Value
**Renal parameters (*N* = 46)**		
Creatinine	0.05 *	0.368
eGFR	−0.66 *	0.33
Urea	0.19	0.10
Uric acid	0.15 *	0.15
**Glycemic profile (*N* = 22)**		
Glycemia	−0.09	0.25
HbA1c	0.35	**0.05**
**Lipid profile (*N* = 41)**		
Total cholesterol	0.03	0.40
HDL-cholesterol	−0.27	**0.04**
LDL-cholesterol	0.18	0.12
Total cholesterol/HDLc ratio	0.28	**0.03**
LDLc/HDLc ratio	0.35	**0.01**
**Calcium and phosphate metabolism (*N* = 46)**		
Calcium	−0.15	0.15
Phosphate	0.05	0.36
Ca × Phosphate product	−0.12	0.22
**Markers of acute inflammation (*N* = 46)**		
Erythrocyte sedimentation rate	−0.03	0.39
Fibrinogen	−0.37	0.40
C-reactive protein	0.30	**0.025**
NLR	0.17	0.14
PLR	−0.14	0.19

Legend: eGFR = estimated glomerular filtration rate; HbA1c = hemoglobin A1c (glycated hemoglobin); HDL = high-density lipoprotein; LDL = low-density lipoprotein; HDLc = HDL-cholesterol; LDLc = LDL-cholesterol; Ca = serum calcium; NLR = neutrophil-to-lymphocyte ratio; PLR = platelet-to-lymphocyte ratio; *p*-values in bold are considered statistically significant; * = Spearman correlation coefficient.

## Data Availability

Data supporting the reported results can be found in the Archive of Clinical Department No. 3, “Carol Davila” University of Medicine and Pharmacy, Bucharest, Romania.
